# The relationship between obesity and nursing care problems in intensive care patients in Austria

**DOI:** 10.1111/nicc.12554

**Published:** 2020-09-20

**Authors:** Franziska Großschädl, Silvia Bauer

**Affiliations:** ^1^ Institute of Nursing Science Medical University of Graz Graz Austria

**Keywords:** BMI, care dependency, intensive care unit, nursing care problems, obesity

## Abstract

**Objectives:**

To describe the characteristics and nursing care problems of intensive care patients in Austria stratified by obesity.

**Background:**

Obese people in intensive care units (ICUs) present nurses with special challenges. Therefore, nurses need to receive education and training regarding how to treat obese patients to provide them with the best care. Most studies on obesity in ICU patients have not specifically addressed the problems and challenges from the nurses' perspective. This may be because nursing science programmes in Europe rarely introduce the topic of obesity.

**Design:**

This was a secondary data analysis of a longitudinal study.

**Methodology:**

The “Nursing Quality Measurement 2.0” is the Austrian version of the “International Prevalence Measurement of Care problems”. It is an annual cross‐sectional study, which has been carried out since 2009. Data from all ICU patients for 2009 to 2018 were extracted and combined into one file (n = 460). The main outcome measures were obesity and various nursing care problems, including care dependency.

**Results:**

Of the ICU patients. 25% were obese. Obese ICU patients suffered significantly more often from diabetes mellitus and endocrine, nutritional, or metabolic diseases than non‐obese patients. About 30% of the ICU patients were totally care dependent, and 85.6% of the ICU patients were at risk of developing pressure ulcers, whereas the risk was higher for non‐obese than obese patients. ICU patients with a risk of pressure ulcer (measured with the Braden Scale) had a reduced risk of being obese (OR = 0.544).

**Conclusion:**

Overall, the prevalence of nursing care problems found in this study was high. No significant differences in the prevalence of nursing care problems between obese and non‐obese patients were found. However, because of the increase in the number of obese patients in all nursing settings, a stronger focus on obesity research in the area of nursing science is recommended.

**What is Known About the Topic**

- Care of obese patients in an ICU is even more demanding.
- Most studies dealing with obesity in ICU patients have not specifically addressed problems and challenges from the nurses' perspectives, which is necessary to improve obesity‐associated nursing outcomes and organizational processes.
- The nursing science programmes in Europe rarely introduce the topic of obesity.

**What this Paper Adds**

- A quarter of the ICU patients investigated was obese.
- The prevalence of nursing care problems among ICU patients was high, but no significant differences between obese and non‐obese subjects were found
- Pressure ulcer risk was identified with lower risk for obesity; this agrees with literature reports that the BMI does not influence pressure ulcers and that being underweight or malnourished is a risk factor for pressure ulcers.

## 1 INTRODUCTION

Obesity is defined as an abnormal or excessive fat accumulation that poses a health risk to those affected. Obesity presents an extremely complex and heterogeneous clinical picture with many different subtypes. It is not caused simply by an imbalance between food intake and energy consumption but also involves environmental factors and genetic aspects.^1^, ^2^ Adults with a body mass index (BMI) of 30 kg/m^2^ and above are classified as obese, whereby the following three classes are distinguished: class 1 (BMI 30.0‐34.9), class 2 (BMI 35.0‐39.9), and class 3 (BMI > 40.0).^1^ According to the World Health Organization,^1^ the prevalence of obesity has tripled globally over the last four decades. In Austria, the prevalence has constantly increased and represents a great public health challenge. In 2014, the age‐standardized prevalence of obesity was 14.6% amongst Austrian adults.^3^

Obese people present health professionals with special challenges. Therefore, health professionals need to receive education and training regarding how to treat obese patients to provide them with the best care. This can prevent treatment problems and delays. A special situation arises when obese patients are admitted to the intensive care unit (ICU). The care of intensive care patients is already complex, cost‐intensive, and poses huge challenges for health workers. However, providing obese patients in an ICU with optimal care is even more demanding because of their obesity‐related pathophysiology,^4^ the disease itself, the severity of the obesity, and obesity‐associated comorbidities. Many secondary diseases can co‐occur with obesity (eg, type 2 diabetes mellitus, heart failure, pancreatitis), and thus, increasing care difficulties arise in ICUs. Obese ICU patients have a higher risk of acute cardiovascular, pulmonary, and renal complications than normal‐weight patients.^2^ They experience longer stays and longer ventilation times in ICUs, as well as a higher number of readmissions to ICUs than non‐obese patients. Overall, obese patients in ICUs require more resources.^5^, ^6^ The care of obese ICU patients requires a higher level of teamwork and an increased use of human resources. Obese ICU patients often receive lower‐quality care because the prerequisites for high‐quality care cannot always be met, diagnostic tests cannot be carried out, and corresponding aids are not available.^4^, ^7^

A phenomenon that has been the subject of extensive research, especially in ICUs, is that a mild or moderate form of obesity has a positive effect on the survival of those affected, while morbid obesity (class 3) seems to be negatively associated with mortality.^8^ This phenomenon (“obesity – survival – paradox”) has been confirmed in several studies.^9^

The nursing profession is the only one whose members are present at the patients' bedsides 24/7. Most studies on obesity in ICU patients have not specifically addressed the problems and challenges from the nurses' perspectives. This may be because nursing science programmes in Europe rarely introduce the topic of obesity.

### 1.1 Study aim

No studies have been conducted on nursing care problems and obesity in ICUs. Therefore, this study was carried out to illustrate the challenges faced by ICU nurses regarding obese patients. These study findings have the potential to help improve obesity‐associated nursing outcomes and streamline organizational processes. The aim of conducting this research was to describe the characteristics and nursing care problems of obese intensive care patients in Austria.

## 2 DESIGN AND METHODS

The study design is a secondary data analysis of a cross‐sectional study. The “Nursing Quality Measurement 2.0” is the Austrian version of the “International Prevalence Measurement of Care problems” (LPZ).^10^ It is an annual cross‐sectional study, which has been carried out since 2009. The study is performed annually on one specific day in different health care organizations in several European countries to collect data on different nursing care problems. For this analysis, only data from ICUs from 2009 to 2018 were used. All patients on an ICU on the day of measurement were asked to participate in the study.

All Austrian health care institutions with more than 50 beds were invited annually to participate in the measurement. A training session was held at the participating institutions to train the nurses about the questionnaires used and the online data entry program. In addition, all training documents were accessible to the participants via a password‐protected area of the website.

### 2.1 Data collection

Two trained nurses from different wards (ie, one from the patient's ward and one from another ward) collected the data together by mainly physically examining the patient. In addition, the patient files could have been used as a source of information. In cases of disagreement, the external nurse's decision was accepted. The original questionnaire was developed based on guidelines and by experts in the Netherlands and tested for different psychometric properties. This questionnaire was translated into German and back‐translated into Dutch by professional translators.^10^ It was used to collect general characteristics and information on different nursing care problems. A secondary data analysis of the general patient characteristics and data on pressure ulcers, falls, and physical restraints was conducted because these data could be compared with data collected from 2009 to 2018.

### 2.2 Variables

The general patient characteristics collected included their age, gender, BMI, medical diagnosis according to ICD‐10,^11^ and level of care dependency.^12^, ^13^ The BMI was measured directly whenever possible or was conveyed by the patient or relatives. The WHO definition of obesity was used^1^ as BMI ≥30 kg/m^2^. Care dependency was measured using the Care Dependency Scale (CDS). This was rated as a valid and reliable tool for ICU patients.^14^ The 15 items of the CDS are assessed on a 5‐point Likert scale. The measurement of pressure ulcers included an assessment of the risk of developing pressure ulcers using the Braden Scale.^15^ Scores between 6 and 23 can be obtained; a score ≤ 20 was considered a pressure ulcer risk. Pressure ulcers were classified according to the National Pressure Ulcer Advisory Panel, European Pressure Ulcer Advisory Panel, and Pan Pacific Pressure Injury Alliance.^16^ A fall was defined as an event where the patients fell to the ground or lower level unintentionally.^17^ All falls that occurred in the 30 days prior to the survey were measured. Physical restraints were defined as measures that restrict human rights and freedom of movement, including all restrictions of personal mobility, such as observation, isolation, manual restraints, and the use of psychological measures.^18^ Physical restraints that had been used in the institution in the 30 days prior to the survey were measured. Data on falls and physical restraints were collected through systematic medical record review.

### 2.3 Data analysis

IBM SPSS Statistics 26 software for Windows was used to perform the data analysis (IBM Corp. Released 2017). Data from all ICU patients for 2009 to 2018 were extracted and combined into one file. We performed descriptive analyses for all variables to determine their distributions and to identify outliers. Metric variables were presented as median and interquartile ranges. Differences between groups were analysed with the chi‐squared and Kruskal‐Wallis tests. A logistic regression analysis was performed with obesity as the dependent variable. Possible influencing variables were identified from an examination of the literature and were analysed as univariate variables. No multicollinearity was assumed if the number of variance inflation factors was fewer than four.^19^ All variables with a *P*‐value lower than .200 were included in the multivariate logistic regression analysis, using the Enter method. Odds ratios were calculated with 95% confidence intervals, and the Hosmer‐Lemeshow goodness‐of‐fit test was used to indicate the fit of the final model. *P*‐values lower than .05 were considered statistically significant.

## 3 RESULTS

### 3.1 General characteristics

Overall, 1030 ICU patients were asked to take part in the measurement, 61.3% of whom gave their informed consent, and 38.7% refused. The main reason for refusal were other reasons (38.1%) followed by being too sick or comatose (33.6%). Patients with no information on BMI (*n* = 171) were excluded from further analysis, resulting in a sample of 460 ICU patients. A total of 25.0% of these patients were obese, with 53.0% of these classified as obesity class I, 27.8% as obesity class II, and 19.2% as obesity class III.

Of the total number of ICU patients, 40.7% were female, and the mean age was 65 years (Table 1). Half of the studied ICU patients had undergone surgery within the 2 weeks prior to the survey. Obese ICU patients suffered significantly more often from diabetes mellitus and endocrine, nutritional, or metabolic diseases than non‐obese patients (*P* < .05). About 30% of the studied ICU patients were totally care dependent.

**TABLE 1:** General characteristics of ICU patients, total and stratified by obesity

	Total ICU patients (*n* = 460)	Non‐obese patients (*n* = 345)	Obese patients (*n* = 115)
Female	40.7%	41.4%	38.3%
Age (median ± IQR)	68 (56‐77)	68 (55‐78)	68 (59‐74)
BMI (median ± IQR)*	26.3 (23.3‐30.0)	24.8 (22.4‐27.1)	33.8 (31.2‐37.9)
Surgery (2 wk prior to survey)	50.0%	51.0%	47.0%
Most prevalent diseases			
Diseases of the circulatory system	57.0%	56.5%	58.3%
Diseases of the respiratory system	30.9%	30.1%	33.0%
Diseases of the digestive system	28.3%	28.7%	27.0%
Diabetes mellitus*	18.9%	15.1%	30.4%
Diseases of the genitourinary system	18.0%	17.1%	20.9%
Endocrine, nutritional, or metabolic diseases*	16.5%	14.5%	22.6%
Number of diseases (median ± IQR)	2 (1‐4)	2 (1‐3)	2 (1‐4)
Care dependency**			
Totally care dependent	29.6%	29.7%	29.6%
To a great extent care dependent	19.6%	20.6%	16.5%
Partially care dependent	20.7%	21.2%	19.1%
To a great extent care independent	17.4%	17.2%	18.3%
Totally care independent	12.6%	11.3%	16.5%
Care dependency (median ± IQR)	45.0 (20.0‐63.0)	44.0 (20.0‐62.0)	46.0 (17.0‐66.0)

Abbreviations: BMI, body mass index; IQR, interquartile range.

* *P* ≤ .05.

** Care Dependency Scale (CDS) was used for the measurement of the level of care dependency. Sum scores ranged from 15 to 75, with a low score indicating a high level of care dependency.

### 3.2 Prevalence of nursing care problems

A risk of developing pressure ulcers was detected for 85.6% of the ICU patients studied, whereas the risk was higher for non‐obese than obese patients (Table 2). The prevalence of restraints did not differ between non‐obese and obese patients. All found differences between obese and non‐obese ICU patients were not significant (*P* > .05).

**TABLE 2:** Prevalence [confidence intervals] of nursing care problems in ICU patients stratified by obesity

	Total ICU patients (*n* = 460)	Non‐obese patients (*n* = 345)	Obese patients (*n* = 115)
Pressure ulcer risk^a^	85.6% [80.3‐88.4]	87.3% [82.5‐90.4]	80.4% [71.4‐87.9]
Pressure ulcers	8.3% [4.3‐9.4]	9.0% [4.6‐11.1]	6.1% [0‐7.7]
Falls^a^	12.4% [8.9‐15.6]	13.4% [9.6‐17.9]	9.5% [3.3‐13.2]
Physical restraints	40.7% [36.4‐46.1]	40.7% [35.0‐46.8]	40.9% [31.9‐53.8]

*Note*: Pressure ulcer risk assessed with the Braden Scale.

a *N* differs slightly because of missing values.

### 3.3 Bivariate and multivariate logistic regression analysis

The bivariate logistic regression analysis yielded two variables with a *P*‐value lower than .200 (Table 3). These variables were included in the multivariate analysis. ICU patients at a risk of pressure ulcer (measured with the Braden Scale) had a 0.544 reduced risk of being obese.

**TABLE 3:** Results of the logistic regression analysis with obesity as the outcome variable (*n* = 460)

	Bivariate		Multivariate	
	*P*‐value	OR [95% CI]	*P*‐value	OR [95% CI]
Gender	.547	0.875 [0.568‐1.349]	—	—
Age	.850	1.001 [0.988‐1.015]	—	—
Surgery	.451	0.850 [0.557‐1.297]	—	—
Number of diagnosis	.160	1.100 [0.963‐1.256]	.133	1.113 [0.968‐1.281]
Care dependency (sum score)	.484	1.004 [0.994‐1.014]	—	—
Risk of pressure ulcer	.076	0.593 [0.333‐1.056]	.043	0.544 [0.301‐0.981]
Pressure ulcer	.331	0.657 [0.281‐1.534]	—	—
Falls	.320	0.677 [0.315‐1.459]	—	—
Physical restraints	.974	1.007 [0.655‐1.548]	—	—

*Note*: Cox & Snell *R* ^2^ 0.012; Nagelkerke *R* ^2^ 0.018; Hosmer‐Lemeshow test *Χ* ^2^ 6.008; *df* = 6; *P* = .422. The non‐occurrence of the problem (no) as reference category. CDS Care Dependency Scale was used for the measurement of the level of care dependency. Sum scores ranged from 15 to 75, with a low score indicating a high level of care dependency.

Abbreviations: CI, confidence interval; OR, odds ratio.

## 4 DISCUSSION

Our study results show the characteristics and nursing care problems of the studied ICU patients in Austria, stratified by obese and non‐obese patients. In our study, the prevalence of obesity is 25%. We found that ICU patients were generally largely dependent on care. A large proportion of ICU patients in this study (85.6%) were at risk of developing pressure ulcers. No significant differences in the prevalence of nursing care problems between obese and non‐obese patients was found. ICU patients with a risk of pressure ulcer had a reduced risk of obesity.

The prevalence of obesity found among ICU patients in our study (25%) is very high compared with the estimated rates in the Austrian adult population (15%). In Australia, the prevalence of obesity in ICU patients is also higher than in the general population. Approximately 75% of the Australian ICU cohort were overweight or obese.^20^ Rosvall et al^6^ reported a prevalence of 36% among ICU patients in Canada. The prevalence of obesity in Austrian ICUs, however, is somewhat lower than that observed in other Western countries, which is in line with the fact that the obesity rate of the Austrian general adult population is also lower than that observed in other industrialized countries.^3^

Furthermore, our study showed that half of the ICU patients were totally or to a great extent dependent on care. Few studies are available on care dependency in ICU patients. Tang et al^21^ showed that 66.4% were dependent in the activities of daily living (ADLs), whereas Guidet et al^22^ found that 27.7% had functional deficits. The differences in these results can be explained by the fact that different assessment tools were used to measure care dependency, functional decline, or ADLs. Our findings and those in the literature on the high degree of care dependency among these patients reveals the burden on caregivers in ICUs; this is even higher when caring for obese ICU patients because many nursing activities (eg, mobilization, redistribution) are made more difficult by high body weight.^4^, ^7^ In addition, functional ability has been shown to be a predictor for 1‐year mortality.^23^ Functional ability and, therefore, care dependency may even worsen after discharge from the ICU.^21^

The literature suggests that incontinence is a particularly relevant problem among ICU patients,^21^ but the number of incontinence studies that include obesity as a potential risk factor is limited.

We found that the pressure ulcer risk in the investigated ICU patients is quite high (85.6%). Results on the prevalence of pressure ulcers in ICUs varied from 33.7%^24^ to 98.7%,^25^ but this variance may also be because of the different risk assessment tools used. In our study, non‐obese patients had a higher risk of developing pressure ulcers and a higher prevalence of pressure ulcers than obese patients. These results agree with those from the logistic regression analysis. Some studies have indicated that BMI does not influence pressure ulcers^26^ and that being underweight or malnourished is a higher risk factor for pressure ulcers.^27^ Nevertheless, pressure ulcers increase the risk of complications,^26^ and therefore, ICU patients should be offered adequate pressure ulcer prevention measures and treatment.

More nursing staff are also required to be able to carry out important prophylactic measures for obese patients, such as changing their position. People with a BMI over 40 kg/m^2^ require at least four carers to carry out appropriate repositioning.^28^ Caregivers experience massive strain while moving and lifting obese people, which can damage their musculoskeletal system and contribute to disorders. In addition, the resources that are necessary to adequately care for obese patients are often not available.^2^ Therefore, nursing practitioners must ensure that suitable bariatric aids for the adequate care of obese patients are available at all times. The routinely used, standardized aids often do not meet the requirements for obese patients and may even severely impair their safety and comfort.^5^

In our study, we found that 40.7% of the studied ICU patients were subjected to standardized physical restraints, with no differentiation made between obese and non‐obese patients. The prevalence rates of physical restraint usage among ICU patients cited in the literature ranges from 23.0%^29^ to 53.9%,^30^ whereas the prevalence rate found in our study (40.7%) falls in the middle. Physical restraints seem to be commonly used to protect medical equipment and subdue unco‐operative patients, although studies have shown that these measures can have many negative consequences.^30^, ^31^ This large variance in the prevalence rates may be because of different time frames (eg, 7 or 30 days prior to the survey) applied to assess the application of physical restraints and the different definitions used.

### 4.1 Limitations

One limitation encountered was that it was not possible to divide the sample into the three obesity classes as the sample comparison groups would have been very small. This limitation may also explain why no significant differences in characteristics and nursing care problems were found. It would be interesting to examine the data by obesity class as the literature indicates that obese people, especially people in obesity class III and up, experience complications more frequently in ICUs. For example, Brodsky et al^32^ noted that intubation can be difficult because of the thick neck circumference, and Ladosky et al^33^ reported that artificial ventilation is often complicated because of the greatly reduced functional residual capacity and heavy chest. These results are mainly from studies conducted in the United States, where the prevalence of obesity class III is high.^34^ The use of a convenience sampling technique resulted in a non‐representative sample. Less than half of the eligible participants could be included in this study. Furthermore, the mechanism of recruitment (those sedated were excluded as being unable to consent to the study) risks the sample being far less likely to be representative of the ICU population in Austria. Nevertheless, a response rate of 61.3% is regarded as satisfactory for an ICU population. The use of the Braden Scale to assess the risk of pressure ulcer also represents minor additional limitations. Results of a recent meta‐analysis show that the Braden Scale had only moderate predictive validity and should be modified for use in ICUs.^35^ Data on falls and physical restraints in the 30 days prior to the survey were collected by reviewing the medical records. In every Austrian health institution, it is obligatory to report physical restraints and falls in the nursing records.^36^ However, especially when the patient is transferred from a prior health institution to an ICU, it could be that these data were not transmitted to the ICU. It is also possible that no medical records will be available when the patient is admitted to an ICU from home. Furthermore, it is not clear what kinds of ICUs participated, an aspect that should be considered in future studies. It would also be interesting to include more variables (eg, duration of hospitalization) to more deeply explore the research question. Unfortunately, other interesting variables were not available, or the collected variables were not comparable for the investigation period. Our study has one prominent strength: For the first time, the nursing care problems experienced in the ICU with obese patients in Austria were systematically examined. Furthermore, in our study, obesity was measured directly, which allowed us to gain more precise insights into this phenomenon compared with previous studies. Our own previous study showed that self‐reported data can deviate significantly from directly measured data.^37^

## 5 CONCLUSIONS

In this study, we found that the prevalence of the investigated nursing care problems was quite high but saw no significant difference between obese and non‐obese ICU patients. We recommend that future researchers investigate this question with large representative samples in ICUs in order to confirm our results. In terms of future research, it would also be interesting to examine other nursing care problems that are potentially related to obesity, such as pain, incontinence, or intertrigo. Findings from these studies could provide important insights that support improvements in nursing care. As the proportion of obese patients increases in all nursing settings, more obesity research in the area of nursing science is needed as well.

### IMPLICATIONS FOR PRACTICE

- Critical care nurses need to be prepared to provide care for obese patients as the number of obese patients is predicted to increase.
- Obesity should be assessed (BMI ≥30) in all patients by the nurses, so they can offer high‐quality care for obese subjects.
- Nurses play a fundamental role in initially identifying and providing advice and care for obese patients early in the treatment process and in helping prevent long‐term negative consequences of obesity.

## AUTHOR CONTRIBUTIONS

All authors have read and approved the paper. **FG**: Conceptualization, data curation, investigation, development of methodology, project administration, writing this paper. **SB**: Data curation, formal analysis, writing this paper.

## ETHICS STATEMENT

All participants or their legal representatives had to sign a written informed consent form. Approval for the study was obtained from an ethical committee (EK number: 20–192 ex 08/09).

## PATIENT CONSENT STATEMENT

All participants or their legal representatives had to sign a written informed consent form.

## Data Availability

Data can be made available if required.
